# On Characterization of Shear Viscosity and Wall Slip for Concentrated Suspension Flows in Abrasive Flow Machining

**DOI:** 10.3390/ma16206803

**Published:** 2023-10-22

**Authors:** Can Peng, Hang Gao, Xuanping Wang

**Affiliations:** 1 State Key Laboratory of High-Performance Precision Manufacturing, Dalian University of Technology, Dalian 116024, China; pengcan2013@gmail.com (C.P.); gaohang@dlut.edu.cn (H.G.); 2 School of Mechanical Engineering, Dalian University of Technology, Dalian 116024, China

**Keywords:** abrasive flow machining, abrasive media, wall slip behavior, capillary flow, shear viscosity

## Abstract

In the realm of abrasive flow machining (AFM), precise finishing and maintaining dimensional accuracy have remained challenging due to non-uniformities in the AFM process and complexities associated with the abrasive media’s shear viscosity and wall slip behavior. By addressing these challenges, this study introduces a comprehensive framework, combining theoretical foundations, measurement techniques, and experimental setups. Utilizing capillary flow, a novel compensation strategy is incorporated within the Mooney method to counter entrance pressure drop effects. This enhanced capillary flow method emerges as a promising alternative to the conventional Cox–Merz empirical rule, enabling precise characterization of wall slip behavior and shear viscosity, particularly at elevated shear rates. The abrasive media exhibit a Navier nonlinear wall slip, as highlighted by the Mooney method. Rigorous verification of the proposed methodologies and models against supplemental experiments showcases a high degree of congruence between predicted and observed results, emphasizing their accuracy and broad application potential in AFM. This research illuminates the intricacies of the abrasive media’s behavior, accentuating the need for meticulous characterization, and provides a robust foundation for genuine modeling and predictions in material removal within AFM.

## 1. Introduction

Abrasive flow machining (AFM) is a non-conventional finishing technology where the abrasive media are driven under pressure to flow back and forth through workpiece surfaces for material removal and surface refinement [1]. These abrasive media are suspensions of abrasive particles dispersed in highly viscous polymer carriers. Due to its excellent machining accessibility, AFM possesses significant advantages in polishing components with intricate or hard-to-access geometries and is widely applied for final surface finishing in the fields of aerospace [2,3], mold fabrication [4], and biomedicine [5].

Despite AFM’s capabilities, achieving precise finishing with maintained dimensional accuracy is challenging. A prevalent issue is the non-uniformity in the AFM process, as evidenced by several studies [6,7,8,9,10,11]. For example, Ferchow et al. [6] and other researchers [7,8,9] reported significant variations in material removal and surface roughness. Our recent findings [10] pinpoint abrupt changes in flow channel geometry as a primary contributor to this non-uniformity. One solution lies in optimizing this geometry, while another approach involves compensating for excessive material removal in certain areas by adding material to these parts [2], and is collectively termed “inverse analysis” of AFM. The success of this inverse analysis heavily relies on authentic modeling and numerical simulation of the abrasive media flow. However, gaps remain in characterizing the abrasive media’s shear viscosity and wall slip behavior.

Given the above challenges, the wall slip phenomenon in AFM requires deeper exploration. This phenomenon, confirmed by numerous studies [2,12,13], refers to the non-zero relative velocity of the abrasive media when in contact with the workpiece wall [14,15]. Wall slip behavior not only serves as a crucial wall/fluid boundary condition but also significantly influences the accuracy and applicability of numerical simulations [13]. The significance of accurately gauging this wall slip velocity is further underscored by its role as a fundamental velocity parameter in material removal models, encompassing the well-known Preston equation [2,16], its variant [10,13], and other comprehensive theoretical models [17,18,19]. In the realm of the wall slip model, Chih-Hua et al. [12] have adopted a linear relationship between shear rate and wall slip velocity. In contrast, a majority of studies have favored a model that explores the nonlinear relationship between wall slip velocity and wall shear stress. This approach is often referred to as the “Navier nonlinear model” [10,13,20,21]. A recurring theme in these studies is their reliance on preset parameters, operating under the assumption that the abrasive media inherently align with these models. Breaking from this trend, Fan et al. [22] uniquely confirmed that the type of abrasive media that exhibited no wall slip on silicone followed the Navier nonlinear wall slip model in AFM. However, their method’s limitation is its applicability only to specific abrasive media.

Given the recognized limitations in current methods for characterizing wall slip behavior, attention has turned to the Mooney method [23], a technique rooted in capillary flow. Commonly used for characterizing wall slip in low-viscosity suspensions, the Mooney method has proven effective in certain scenarios [24,25]. However, its applicability to the high-viscosity abrasive media in AFM remains a gray area. A limitation of the Mooney method is its propensity to overlook the entrance pressure drop in capillary flow, assuming that the fluid remains fully developed across the total capillary [26]. While such an assumption might be tenable for low-viscosity fluids, it may falter when dealing with the abrasive media which boasts highly viscoelastic properties. Given this backdrop, there is an imperative factor in the entrance pressure drop and to architect a holistic framework tailored for the wall slip characterization of the abrasive media.

Beyond wall slip, grasping the shear viscosity–shear rate relationship of the abrasive media is pivotal for its constitutive model, which is fundamental to accurate flow field simulations in AFM. While rotational rheometers offer a direct means to measure the shear viscosity of the abrasive media, the presence of wall slip of the abrasive media complicates matters. At high shear rates, wall slip or even sample extrusion occurs on the testing fixtures [27,28], leading to measurements that deviate significantly from theoretical values [29,30]. The current strategy among researchers to circumvent this issue is to employ small amplitude oscillatory shear (SAOS) tests and the Cox–Merz rule [31] to derive the shear viscosity–shear rate relationship [32,33]. This rule suggests that a fluid’s shear viscosity and shear rate can be represented by its relationship between complex viscosity and angular frequency in SAOS tests [31]. However, this approach is only valid for the abrasive media that adhere to the Cox–Merz empirical rule. It is worth noting that capillary rheometry can compensate for the measurement errors induced by wall slip in the abrasive media. Nevertheless, for the abrasive media with higher abrasive particle mass fractions and larger abrasive sizes, which result in test samples on a millimeter scale, there are hardly any commercial capillary rheometers that meet the measurement demands. Thus, a capillary flow-based apparatus to measure the viscosity of the different abrasive media is urgently needed. Such a development holds promise for facilitating genuine modeling and accurate predictions of material removal.

Given these challenges, this research aims to establish a framework for measuring the shear viscosity and wall slip behaviors of the abrasive media in AFM. This involves establishing a solid theoretical and experimental foundation to effectively address two main issues associated with the AFM process. The approach is groundbreaking as it merges, for the first time in AFM, the principles of capillary flow and the Mooney method while also correcting the entrance pressure loss. The Mooney method effectively compensates for the impact of wall slip of the abrasive media on the measurement results when using capillary flow for shear viscosity measurement. Given the high viscosity of the abrasive media, the correction for entrance pressure loss is expected to further enhance the measurement accuracy. The method involved experimental evaluations of the two typical abrasive media and theoretical assessments of two viscous constitutive models, thereby holding promise for verifying the adaptability of the proposed method.

## 2. Theoretical Analysis for Determining Shear Viscosity and Wall Slip Behaviors Using Capillary Flow Test

In this research, we introduced the capillary flow test (CFT) as an extended approach for assessing the wall slip behavior and the connection between the shear viscosity and shear rate of the high-viscosity abrasive media. The strength of this method lies in its ability to conduct measurements under real flow conditions, eliminating the necessity for specialized treatment of the abrasive media.

**Figure 1:**
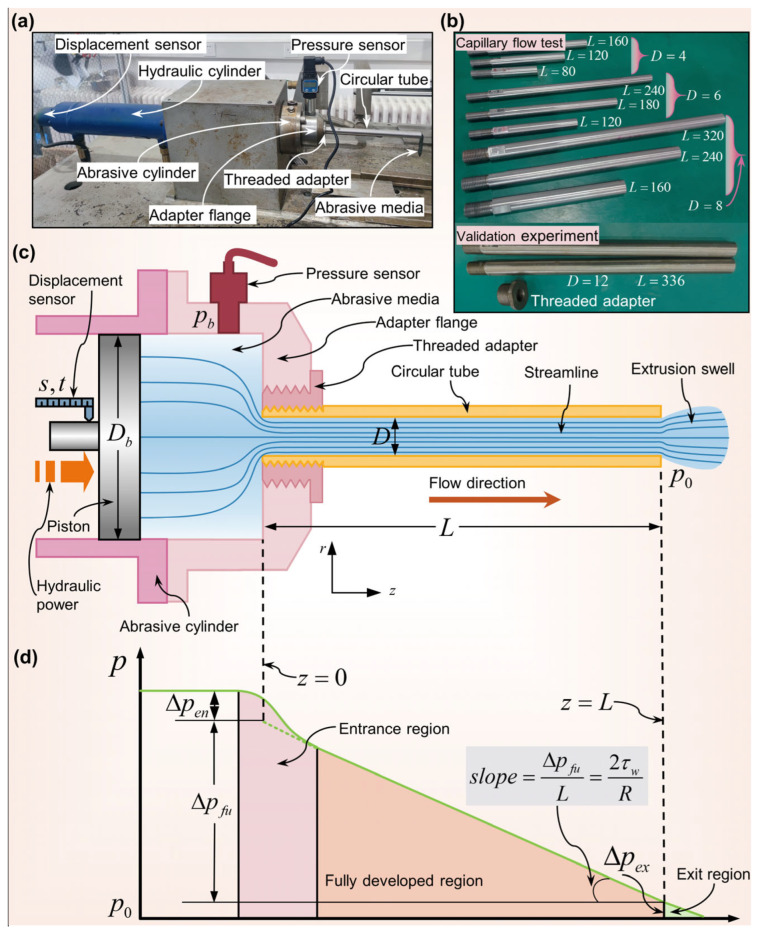
Capillary flow experimental setup and schematic. (**a**) A photograph of the experimental setup, (**b**) circular tubes of various diameters and aspect ratios, (**c**) a schematic representation of the experimental setup, and (**d**) a schematic showing the variation of pressure along the flow direction in capillary flow, highlighting the distribution in the entrance, fully developed region, and exit areas.

Figure 1c,d show our method, which extrudes the abrasive media from a large-diameter barrel into a capillary tube, or a circular tube, with diameter D and length L. Within the circular tube, the flow of the abrasive media can be divided into three regions: the entrance, the fully developed, and the exit region.

**Figure 2:**
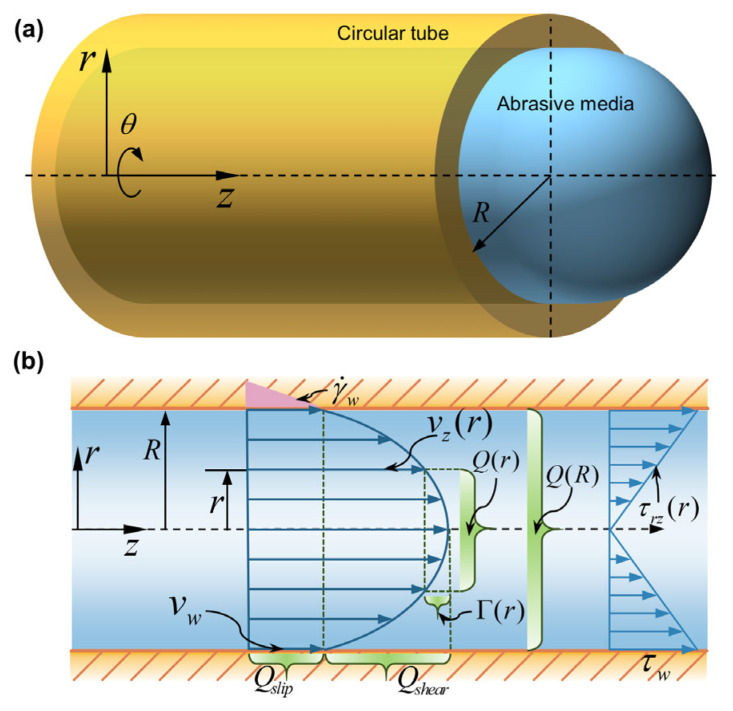
Laminar flow dynamics in a circular tube: (**a**) Diagram illustrating the cylindrical coordinate system utilized for the flow of abrasive media. (**b**) Depiction of the velocity profile and shear stress distribution under conditions of fully developed, steady-state flow with a wall slip.

At a macroscopic level, we can consider the abrasive media as a homogeneous fluid in continuous flow. Due to its high viscosity (on the order of 100–1000 Pa∙s), the flow in the fully developed region exhibits the characteristics of steady-state, isothermal laminar flow, as the Reynolds number calculated is less than 1. Considering wall slip of the abrasive media, Figure 2 depicts its velocity profile and wall shear stress distribution in the fully developed region of capillary flow. Here, the pressure gradient (∂p/∂z and wall shear stress $\tau_{w}$ are constant in the flow direction, while the wall velocity $v_{w}$ is non-zero. In the cylindrical coordinate system (r, θ, z), the shear stress $\tau_{rz}$ is zero at the tube center, increases proportionally with the radius r, and peaks at the wall (see Figure 2b), i.e.,

(1) $$\tau_{rz}(r)=-\frac{r}{2}\frac{\partial p(r,z)}{\partial z}$$

The shear rate $\dot{\gamma }$ is defined as the negative value of the velocity gradient $-dv_{z}(r)/dr$ and is solely dependent on the shear stress $\tau_{rz}$. This relationship between $\dot{\gamma }$ and $\tau_{rz}$ is an inherent rheological characteristic of the abrasive media, often described by its constitutive equation. Hence, the volumetric flow rate Q(r) in the virtual core flow with a radius r (refer to Figure 2b) is achieved by integrating the product of velocity and the area element 2πrdr across r as follows:

(2) $$Q(r)=2\pi \int_{0}^{r}v_{z}(r)rdr$$

By partially integrating Equation (2) and transforming the independent variable from radius r to shear stress $\tau_{rz}$, we obtain

(3) $$Q(r)=\pi r^{2}v_{z}(r)+\frac{\pi r^{3}}{\tau_{w}{}^{3}}\int_{0}^{\tau_{rz}(r)}\dot{\gamma }(\tau_{rz})\tau_{rz}{}^{2}d\tau_{rz}$$

In the above equation, we denote the second integral function on the right side as $\Gamma (\tau_{rz})$. Therefore, in the context of wall slip, the volumetric flow rate of the abrasive media’s fully developed steady-state laminar flow fulfills the following relationship:

(4) $$Q(R)=Q_{slip}+Q_{shear}=\pi R^{2}v_{w}+\Gamma (\tau_{w})$$

where $v_{w}$ stands for the wall slip velocity. As evident from Equation (4), the equation’s right-hand side’s first term represents the plug flow created by the wall slip velocity, while the second term signifies the volumetric flow rate due to pressure-driven flow in the absence of wall slip.

The apparent shear rate in capillary flow is defined as ${\dot{\gamma }}_{a}=4Q(R)/(\pi R^{3})$. Upon substitution into Equation (4), we obtain

(5) $${\dot{\gamma }}_{a}=\frac{4v_{w}}{R}+\frac{4}{R}\frac{\Gamma (\tau_{w})}{\pi R^{2}}$$

According to the assumptions made by Mooney [23], the fluid’s wall slip velocity is solely linked to the wall shear stress. Since the second term on the right side of Equation (5) is merely dependent on the shear viscosity traits ${\dot{\gamma }}_{rz}(\tau_{rz})$ of the abrasive media, the apparent shear rate turns into a function of the capillary radius R under a fixed wall shear stress. Thus, the partial derivative of Equation (5) with respect to 1/R gives

(6) $$v_{w}\left(\tau_{w}\right)={\frac{\partial {\dot{\gamma }}_{a}}{4\partial (1/R)}|}_{\tau_{w}=\mathrm{constant}}$$

Hence, considering the apparent shear rates at various tube diameters under the same wall shear stress, if the relationship between 4/R and ${\dot{\gamma }}_{a}$ yields a straight line, it implies that the assumption that the wall slip velocity is solely related to the wall shear stress holds. Moreover, the slope of this line equates to the wall slip velocity $v_{w}(\tau_{w})$. This technique of calculating the wall slip velocity is known as the Mooney method [23]. If wall slip is absent, the left side of the equation equals zero, implying that the $\tau_{w}$−${\dot{\gamma }}_{a}$ curves of tubes of different diameters coincide. Therefore, the presence of wall slip in the flow of the abrasive media can be discerned by examining the diameter dependence of the $\tau_{w}$−${\dot{\gamma }}_{a}$ curves.

When wall slip is present, to determine the relationship between $\tau_{w}$ and ${\dot{\gamma }}_{w}$ for understanding the shear viscosity characteristics of the abrasive media, we refer to the Weissenberg–Rabinowitsch (W−R) correction [34] for the wall shear rate of capillary flow in the absence of wall slip. Differentiating Equation (5) with respect to the wall shear stress $\tau_{w}$ and by rearranging it, we obtain

(7) $${\dot{\gamma }}_{w}=({\dot{\gamma }}_{a}-\frac{4v_{w}}{R})\left[\frac{3}{4}+\frac{1}{4}\frac{d\ln({\dot{\gamma }}_{a}-\frac{4v_{w}}{R})}{d\ln\tau_{w}}\right]$$

The true wall shear stress can be obtained by plotting $\ln({\dot{\gamma }}_{a}-4v_{w}/R)$ against $\ln\tau_{w}$ and using this equation.

In summary, determining the pressure gradient in the fully developed region of capillary flow, as represented by the slope of the straight line in Figure 1d, is pivotal to analyzing the rheological and wall slip behavior of the abrasive media. However, as shown in Figure 1d, the pressure drop during capillary flow is tripartite: there is an initial pressure loss resulting from sudden contraction in the entrance region, a linear decrease observed in the fully developed region, and a typically non-zero pressure at the exit attributed to the elastic energy storage and viscous energy dissipation inherent in the abrasive media. When the length-to-diameter ratio L/D of the capillary is substantial, the pressure drop $\Delta p_{ex}$ at the exit becomes insignificant in comparison to the overall pressure drop, or the extrusion pressure $p_{b}$, and can thus be ignored. Considering the viscoelastic nature of the abrasive media, it is imperative to note that the entrance pressure loss is non-trivial. Therefore, the equivalent pressure drop $\Delta p_{fu}$ in the fully developed region of the abrasive media across the capillary’s entire length is derived from $p_{b}$ minus the entrance pressure loss $\Delta p_{en}$, which can be expressed as

(8) $$p_{b}=\frac{4L}{D}\tau_{w}+\Delta p_{en}$$

This equation signifies that, given the same apparent shear rate, the total pressure drop of capillary flows comprise varying lengths; however, identical diameters linearly relate to 4L/D. By fitting this linear relationship, the slope of the fitted line is the wall shear stress $\tau_{w}$, and the intercept extrapolated to 4L/D=0 equals the entrance pressure loss $\Delta p_{en}$ at that apparent shear rate. This process of correcting the entrance pressure loss to account for end effects is known as Bagley’s correction [35]. With this method, it is possible to focus solely on measuring the pressure inside the abrasive cylinder $p_{b}$, eliminating the need for measurements past the contraction zone. Crucially, the Bagley correction remains accurate regardless of the presence of stagnation zones or viscoelastic flows. This is because, in a contraction flow channel of a specific geometry, the entrance pressure loss, given a certain inlet flow rate, is predominantly influenced by the fluid’s rheological properties.

In conclusion, the rheological and wall slip behaviors of the abrasive media can be comprehensively characterized by integrating the Bagley correction, the Mooney method, and the W−R correction. This characterization involves several specific steps:

(1) Measuring the flow rate and total pressure drop across capillaries with different length-to-diameter ratios and diameters within a designated flow rate range.
(2) Applying the Bagley correction to these measurements to discern the dependency of $\tau_{w}$ on ${\dot{\gamma }}_{a}$ for various diameters.
(3) Utilizing the Mooney method (as per Equation (6)) to establish the correlation between wall slip velocity and wall shear stress.
(4) Employing Equation (7) to derive the relationship between shear viscosity and shear rate.

**Figure 3:**
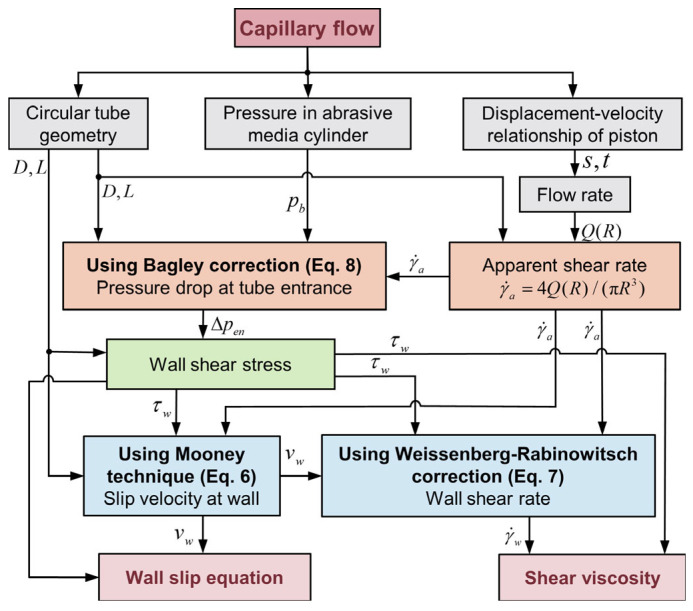
Flowchart for rheological and wall slip characterization of abrasive media using CFT: A step-by-step visualization of the integrated approach combining the Bagley correction, Mooney method, and W−R correction.

For clarity, the entire solution process was encapsulated in Figure 3.

## 3. Experimental Materials and Methodology

### 3.1. Preparation of Abrasive Media

**Figure 4:**
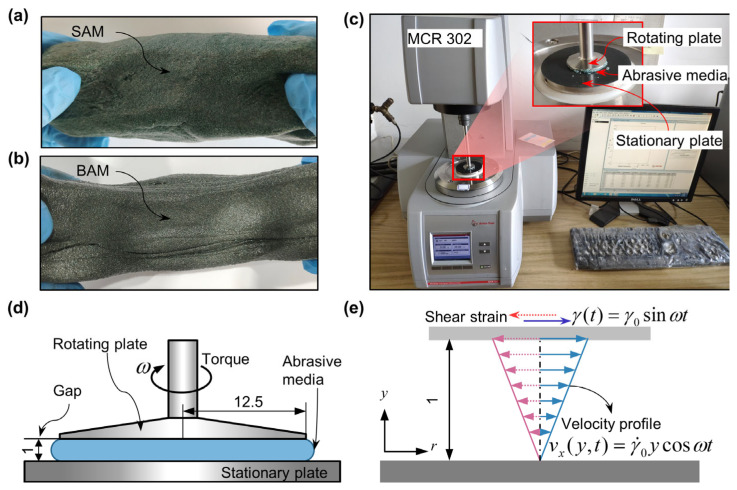
Presentation of abrasive media used in the experiment and the rotational rheometer: photograph of (**a**) SAM and (**b**) BAM; (**c**) real image of the parallel plate measuring fixture with a local magnification; (**d**) schematic of the parallel plate measuring fixture; (**e**) illustration of the alternating shear strain γ(t) and velocity profile $v_{x}$ on the abrasive media during small amplitude oscillatory shear. All dimensions are denoted in millimeters.

In this study, two types of abrasive media were utilized to validate the adaptability of the method for assessing wall slip behavior and rheological properties. As illustrated in Figure 4a,b, these media use matrix materials of styrene-butadiene rubber (SBR) [36,37,38] and boron-modified silicone rubber (BSR). Specifically, the SBR-based abrasive media is termed SAM, while BSR-based abrasive media is labeled BAM.

The preparation of these abrasive media consisted of two primary stages: plasticization of the polymer matrix and subsequent mixing. Initially, plasticizers were added to each prepared matrix, turning the solid polymers into fluid-like substances. Lubricants and 80# green silicon carbide abrasive particles were then incorporated, and the mixtures were homogenized using a stirrer. After this, the mixture was allowed to stand undisturbed for over 120 h, ensuring the dissipation of any air bubbles formed during mechanical stirring, thereby minimizing their potential impact on experimental results. To control for the influence of abrasive particle quantity in the media, both abrasive media types were formulated to have an equivalent volume fraction of abrasive particles. More specific parameters can be found in Table 1.

### 3.2. Capillary Flow Setup

Capillary rheometers, typically used to gauge the steady-state shear rheological attributes of polymer melts, are also instrumental in probing wall slip behavior related to shear stress. In our study, the abrasive media, with a particle size exceeding 0.1 mm and a volume fill rate over 10%, was classified as a dense suspension fluid. This classification presented a significant challenge. Standard capillary rheometers, conforming to ISO 11443-2021, are equipped with capillaries having diameters ranging from 0.5 mm to 2 mm. Such dimensions can obstruct the flow of our particular abrasive media, with finer diameters risking accuracy due to the abrasive wear effect.

Addressing this concern, we designed a capillary rheometer apparatus specifically tailored for the abrasive media’s wall slip behavior assessment. As depicted in Figure 1a,c, this apparatus integrates a pressure measurement module with a horizontally oriented AFM instrument. Its design includes the following:

(1) An abrasive cylinder of 50 mm diameter and 220 mm length.
(2) 304 stainless steel tubes of different specifications, detailed in Figure 1b.
(3) A hydraulic system.
(4) Two specialized sensors: a flat-film pressure sensor strategically placed close to the constriction (specifications: CYYZ51X, ranges 0–2.5 MPa and 0–10 MPa; repeatability: 0.1% F.S.; from Star Sensor Manufacturing Co., Ltd., Langfang, China); and a magnetostrictive displacement sensor (specifications: BYDS-OPLC03VM1-A3010G200FO; repeatability: ±0.002% F.S.; from Shanghai Boyi Industrial Co., Ltd., Shanghai, China).
(5) An adapter flange making a converging half angle of 90° to the horizontal, aiming to achieve uniform pressure in the abrasive cylinder.

All the steel tubes were precision-grounded to meet specific dimensions, adhering to a tolerance of ±0.005 mm. For our experiments, tubes of diameters 4 mm, 6 mm, and 8 mm explored the abrasive media’s wall slip behavior. Conversely, a tube with a 12 mm diameter and 336 mm length was employed to validate both the wall slip behavior and rheological characterization. Initially, they maintained an inner wall roughness of about Ra 1.6 μm. After experimentation, this surface roughness was reduced to Ra 0.8–0.9 μm. While there is a noticeable reduction in the inner wall roughness, for the purpose of simplifying our study, we have chosen to overlook the potential influence of this roughness on wall slip behavior.

During the experimental trials, the abrasive media were extruded from the cylinder under ambient conditions. Due to the duration of the experiments and unavoidable seasonal temperature fluctuations, the tests for SAM were conducted at 20 °C, while those for BAM were performed at 15 °C. It is worth noting that the primary focus of this study is not to investigate the impact of temperature on the experimental results. Moreover, the studies on slip behavior and shear viscosity for both abrasive media were conducted independently. While we acknowledge the temperature inconsistency between the two sets of trials, prior literature [13,36] and preliminary tests have indicated that such a minor temperature difference is unlikely to introduce significant variation in the wall slip behavior and shear viscosity of the abrasive media under investigation. Nevertheless, future studies aiming for a comprehensive understanding could consider maintaining a consistent temperature environment.

To guarantee wall slip of the abrasive media within the tube, the extrusion pressure $p_{b}$ was carefully regulated at 1–9.5 MPa. Throughout this phase, internal cylinder pressure and piston displacement s were meticulously recorded. A steady-state flow was inferred when the piston’s displacement demonstrated linear progression and the pressure remained stable. The extrusion pressure during this steady state was deduced by averaging the data from the pressure sensor. The piston’s velocity was determined by performing a linear fit on the displacement–time s−t data collected during this phase. Multiplying the piston’s velocity by its cross-sectional area gave us the volume flow rate.

To ensure the reliability and accuracy of the experimental results, each pressure-specific capillary flow test was repeated three times, and the averaged value from these iterations was accepted as the conclusive data. To prevent viscosity drops from temperature rises after multiple extrusions, we discarded the used abrasive media.

Additionally, a pre- and post-test diameter assessment of every stainless steel tube was instituted. These measurements revealed that the post-test diameter increase never exceeded 0.01 mm. Given the minuscule nature of this increment, we inferred that its bearing on our results was negligible. This led to the assumption in our subsequent data analyses that all tubes maintained a consistent diameter.

### 3.3. Small Amplitude Oscillatory Shear and Controlled Shear Rate Tests

The Anton Paar MCR302 rotational rheometer, equipped with a 25 mm diameter parallel plate, as shown in Figure 4c,d, was employed to assess the validity of the Cox–Merz rule for the two abrasive media used in our study. We utilized two distinct testing modes of the rheometer: small amplitude oscillatory shear (SAOS) and controlled shear rate tests (CSRT). The SAOS mode, often employed in rheological studies, offers insights into the dynamic rheological behavior of materials without inducing structural breakdown. On the other hand, CSRT directly measures the flow characteristics of materials under specified shear conditions. It is worth noting that while the data from the rotational rheometer might be skewed at higher shear rates due to wall slip, they remain reliable at lower shear rates where such slip is minimal or non-existent. For our study, both tests were crucial: the SAOS test was conducted to determine if the abrasive media conformed to the widely recognized Cox–Merz rule, and the CSRT provided a direct measure of their flow properties. During testing, we maintained a consistent plate gap of 1 mm. Given the ambient temperatures observed during CFT with SAM and BAM, which were 20 °C and 15 °C, respectively, the rheometer’s testing temperature was calibrated to match these specific conditions.

Before diving into the SAOS measurements, we performed an amplitude sweep set at an angular frequency ω of 10 rad/s. This procedure was pivotal in delineating the linear response relationship between both the storage modulus $G^{\prime }$ and the loss modulus $G^{\prime \prime }$ with ω. Based on the amplitude sweep results, the shear strain amplitude $\gamma_{0}$ for SAM was determined to be 1%, while for BAM it was 0.1%. In the SAOS phase, the abrasive media samples were subjected to shear strains, as graphically represented in Figure 4e, allowing us to gauge their shear stress reactions. The ω spanned a range of 0.1–628 rad/s. Throughout this process, key parameters, such as $G^{\prime }$, $G^{\prime \prime }$, complex viscosity |η*|, and the damping factor tanδ, were meticulously recorded. The equation defining complex viscosity |η*| is as follows:

(9) $$\left|\eta *\right|=\frac{\sqrt{{G^{\prime }}^{2}+{G^{\prime \prime }}^{2}}}{\omega }$$

The parameter tanδ, which symbolically represents a fluid’s energy dissipation, is derived from the ratio of the fluid’s loss modulus to its storage modulus as follows:

(10) $$\tan\delta =\frac{G^{\prime \prime }}{G^{\prime }}$$

Interpreting the value of the damping factor is insightful: a value less than one (tanδ<1 ) signals the prevalence of the material’s elastic behavior, whereas a value exceeding one (tanδ>1) indicates a more pronounced viscous behavior.

For the CSRT, determining an appropriate shear rate range is pivotal. It not only ensures that we capture the shear rate regions where the abrasive media do not exhibit wall slip but also prevents us from venturing into excessively broad testing ranges, which might yield irrelevant data due to excessive wall slip. Guided by these considerations and preliminary experiments, we determined a shear rate range of 0.01–10 s^−1^ for our tests. To strike a balance at measurement points throughout the range, the shear rate was modulated logarithmically. We dedicated a timeframe equivalent to double the inverse of the shear rate for each measuring point, thereby ensuring heightened accuracy, especially at the lower end of shear rates. As the rotational tests progressed, both shear stress and shear viscosity were rigorously monitored and recorded.

## 4. Results and Discussion

### 4.1. Analysis of Capillary Flow Results and Discussion on Wall Slip Models

#### 4.1.1. Entrance Pressure Loss Correction

This section focuses on the exploration of the capillary flow performance exhibited by the abrasive media of SAM and BAM, with a special emphasis on understanding their extrusion pressures, wall shear stresses, and the intricate dynamics of entrance pressure losses.

**Figure 5:**
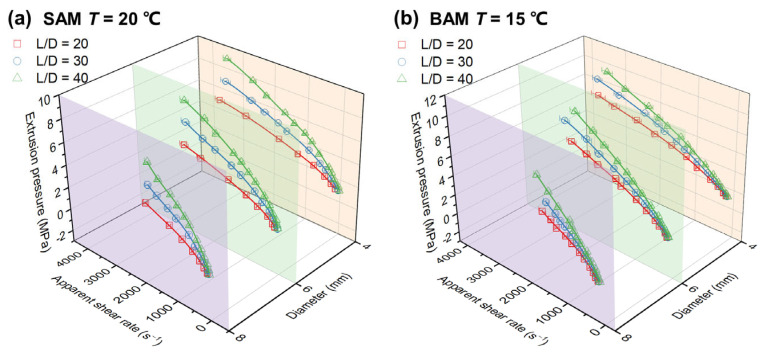
Three-dimensional scatter and line plots of extrusion pressure vs. apparent shear rate for (**a**) SAM and (**b**) BAM, showcasing dependency on capillary diameter and length-to-diameter ratio. Experimental data points with error bars represent standard deviations in pressure and shear rate, with different colors and shapes signifying varying length/diameter ratios. Transparent planes of different colors represent the diameter of the capillary. Interpolated curves derived from cubic B-spline interpolation highlight the trends.

**Figure 6:**
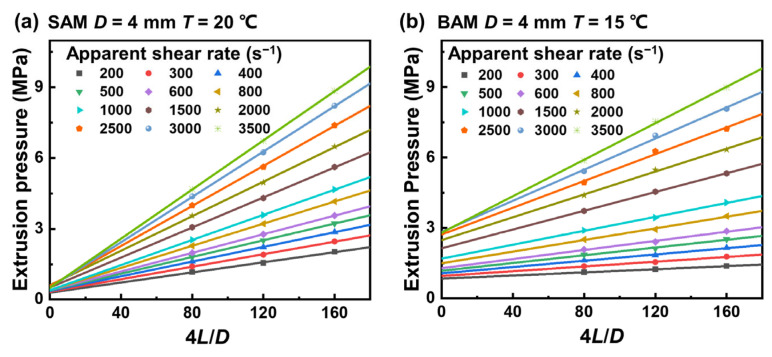
Bagley correction plots comparing the flow behavior of (**a**) SAM and (**b**) BAM in a capillary with a diameter of 4 mm. The data points represent the results obtained from the cubic B-spline interpolation in Figure 6, with differences in color and symbol shape indicating different apparent shear rates. Notably, only a subset of the apparent shear rates are shown for clarity. The straight lines represent the linear relationship between the extrusion pressure and 4L/D in capillaries with the same diameter and apparent shear rate but different aspect ratios. The slopes of these lines represent the wall shear stress, and the intercepts on the y-axis represent the entrance pressure drop in capillary flow.

Derived from the relationship between extrusion pressure and volume flow rate in relation to the apparent wall shear rate, the total pressure drop is detailed in Supplementary Tables S1–S6. Using this foundational data, Figure 5 was constructed to show the connection between extrusion pressure, apparent shear rate, diameter, and aspect ratio. Figure 5a shows that, with a consistent tube diameter, there is an increase in extrusion pressure as the apparent shear rate increases, although the rate of increase gradually diminishes. Similarly, the capillary flow dynamics of BAM, as shown in Figure 5b, follow a comparable pattern, confirming the shear-thinning tendencies inherent to both abrasive media. Interestingly, at the same diameter, SAM’s extrusion pressure rises along with the aspect ratio more than BAM. This suggests that SAM’s pressure drop in the fully developed region is more noticeable, as supported by Figure 6.

To reduce the potential effect of the entrance’s pressure drop on the relationship between pressure drop and flow rate in the fully developed region, which could distort the interpretation of experimental results, we applied a cubic B-spline interpolation to the collected data. This analytical approach is culminated in the Bagley correction plots for both SAM and BAM, with the results for a 4 mm diameter shown in Figure 6a,b. Additional data for tubes with diameters of 6 and 8 mm can be found in Figure S1 in the Supplementary Materials. Based on Equation (8), the slope of the linear fit, at a given apparent shear rate, equals the associated wall shear stress. Notably, the data shows a nearly perfect linear relationship for the given shear rates. This supports the effectiveness of using the Bagley correction to calculate the entrance region’s pressure loss and wall shear stress.

**Figure 7:**
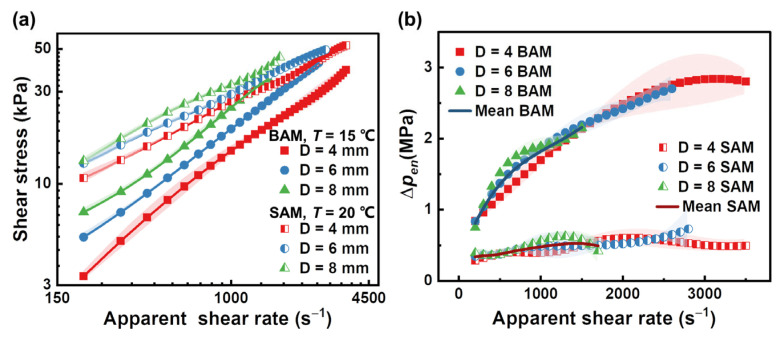
Dependence of (**a**) the wall shear stress versus apparent shear rate relationship on a logarithmic scale, and (**b**) the entrance pressure loss versus apparent shear rate relationship on a linear scale, on the diameter of the capillaries. The data are derived from the capillary flow Bagley correction for both SAM and BAM. Different colors and shapes of the symbols represent different capillary diameters, where half-filled and solid symbols correspond to SAM and BAM, respectively. Standard deviations from the Bagley correction are depicted through error bands matching the color of the corresponding symbols. The curves in (**a**) represent cubic B-spline interpolations of the data points, which will be used for subsequent Mooney method analysis. The curves in (**b**) represent the average values of the entrance pressure loss, which will be used for predicting the total pressure drop.

Figure 7a, presented in logarithmic format, reveals the impact of capillary diameter on the relationship between wall shear stress and apparent shear rate for our abrasive media, based on the Bagley correction. The plot indicates that the flow patterns for both SAM and BAM are significantly influenced by the diameter. At a set apparent shear rate, there is a clear increase in wall shear stress in line with a growth in tube diameter. From the discussion in Section 2, this highlights the presence of wall slip phenomena in both abrasive media. It suggests the relevance of considering slip during the interaction at the tube wall/fluid boundary. Such findings stress the importance of considering the wall slip phenomenon when evaluating and improving the AFM polishing process because of its potential effect on the velocity distribution of the abrasive media and, as a result, the polishing quality.

Additionally, the y-intercept during the Bagley correction process represents the entrance pressure loss for a particular shear rate. Figure 7b displays this entrance pressure loss in relation to the apparent shear rate, depending on the diameter and abrasive media. Clearly, BAM has a greater entrance pressure loss than SAM at the same apparent shear rate. Also, the rise in entrance pressure loss for BAM is more pronounced than that for SAM as the apparent shear rate increases. These differences might come from the unique linear viscoelastic properties of the abrasive media, which will be covered in greater detail in Section 4.2.1. This noticeable increase in the entrance pressure loss also highlights the importance of the Bagley correction.

#### 4.1.2. Wall Slip Behavior Analysis of SAM and BAM

In this section, we direct our attention to the wall slip behavior exhibited by both SAM and BAM. Our discussion encompasses the alignment of various slip models with the observed experimental results, along with a preliminary exploration into the mechanisms and determinants underpinning wall slip.

**Figure 8:**
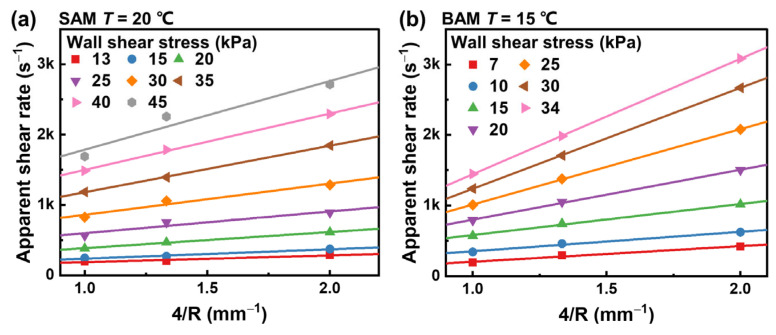
Mooney plots of (**a**) SAM and (**b**) BAM. The data points are derived from cubic B-spline interpolation in Figure 7a, with different colors representing different shear stresses. The straight lines represent linear fits to the data points, the slopes of which equal the wall slip velocities. Only a subset of the wall shear stresses are shown for clarity.

We applied cubic B-spline interpolation to the data from Figure 6, resulting in the Mooney plots shown in Figure 8, which correspond to specific wall shear stresses. We also performed a linear fit on this figure. Based on Equation (6), the slope of this fitted line stands as an indicator of the wall slip velocity that corresponds to the wall shear stress. It is worth highlighting that, while a few isolated data points veer off from the fitted line, the majority of the experimental data stays in close alignment with this line. This alignment underscores the Mooney method’s effectiveness in deducing the wall slip velocity of the abrasive media. Furthermore, a noticeable trend emerges. As the wall shear stress rises, the slope of the fitted line sees a corresponding increase, suggesting a positive relationship between wall shear stress and wall slip velocity.

**Figure 9:**
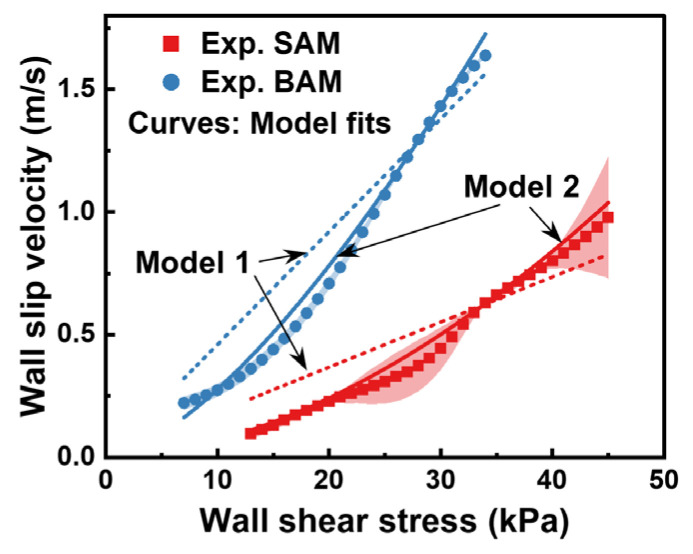
Comparison of wall slip behaviors for SAM and BAM. The scatter plot with error bands illustrates the results from the Mooney method. The curves represent the fitted values based on the Navier linear slip model (Model 1) and the Navier nonlinear slip model (Model 2), with the 95% confidence interval indicated for each.

For a clearer visualization of the dynamics between wall slip velocity and wall shear stress, we turn to Figure 9. Here, we utilized two models, the Navier linear model (termed as Model 1) [39] and the Navier nonlinear model (termed as Model 2) [15], to delineate the experimental relationship between the two parameters. Both models share the following mathematical representation:

(11) $$v_{w}=k_{s}\tau_{w}{}^{m_{s}}$$

In this equation, $k_{s}$ represents the wall slip coefficient, a measure of the slip’s intensity. A value of $k_{s}=0$ indicates a scenario where there is no slip at the boundary. On the other hand, $m_{s}$ is termed the slip exponent. A value of $m_{s}=1$ portrays a scenario where wall slip velocity shares a direct proportionality with the shear stress, which is characteristic of the Navier linear model.

During our fitting procedure, we prioritized data points with smaller errors by assigning them more weight. This strategy was adopted to minimize the influence of data points with larger inaccuracies, leading to a representation that mirrors the real trend of the data more reliably. The fit of the models was evaluated through metrics such as the coefficient of determination (R^2^), the Akaike information criterion (AIC), and the Bayesian information criterion (BIC). We have listed the fitting parameters along with the results pertaining to the model’s fit quality in Table 2. A clear pattern emerges from the data: for both SAM and BAM, Model 2 consistently showcases an R^2^ value closer to 1 and more favorable AIC and BIC values. This suggests a superior fit of the Navier nonlinear slip equation for describing the slip behavior of our subjects, SAM and BAM. These findings resonate with those observed by Fan et al. [22]. They reported a slip exponent $m_{s}$ that changed between 1.41 and 1.62, depending on the wall’s roughness, and a slip coefficient magnitude between 10^−6^ and 10^−5^ $\mathrm{mm}/s\cdot {\mathrm{Pa}}^{-m_{s}}$. These figures mirror our findings. However, our method offers greater adaptability compared to their study.

An interesting observation for BAM was that the R^2^ for Model 1 scaled up to a notable 0.924. This discovery reveals that although the nonlinear model performs excellently in most scenarios, the linear model remains a viable choice when the degree of wall slip is significant. This is further reinforced by the work of Chih-Hua et al. [12]. They adopted a wall slip model similar to our linear model, and the results from their simulations showed a commendable match with experimental observations.

Another important observation merits discussion. Despite the experimental data aligning well with Model 2’s fitting curve, as illustrated in Figure 9, discrepancies still exist in certain regions, such as in the case of BAM when the wall shear stress lies between 20 and 30 kPa. Two potential reasons can be identified. Firstly, the constant pressure type capillary rheometer used in our experiments necessitated the use of the Bagley correction through the cubic B-spline interpolation, which might have introduced errors related to the analytical method. Secondly, our model might have overlooked other variables that could influence wall slip velocity. For instance, the potential reduction of the capillary’s inner wall roughness by the abrasive media during experimentation was not factored in. This change might noticeably affect wall slip behavior.

#### 4.1.3. Wall Slip Mechanism and Lubricant’s Effects on Slip Behavior

This section explores the wall slip mechanism of the abrasive media and analyzes the effects of a lubricant on slip behavior.

A comparative analysis of the wall slip velocities of SAM and BAM under the same wall shear stress conditions revealed a distinct pattern. Across our experimental domain, BAM consistently had higher slip velocities compared to SAM, emphasizing its enhanced wall slip properties.

**Figure 10:**
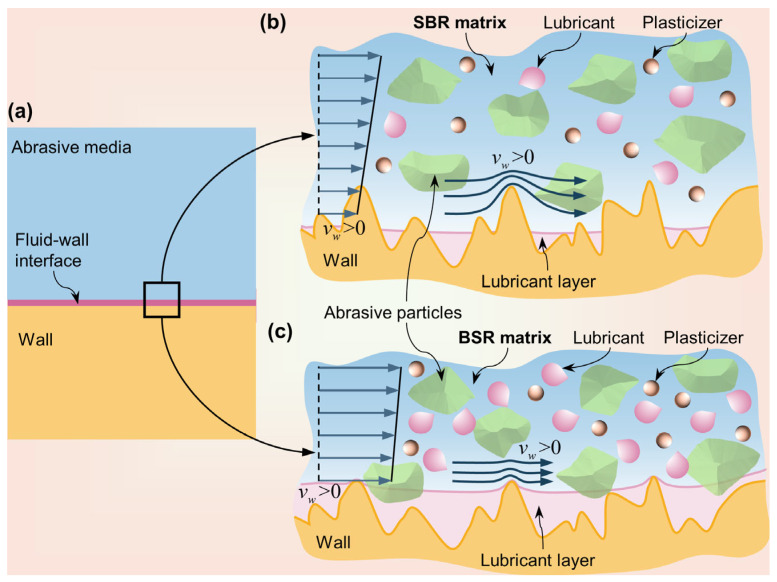
Microscopic schematic of the abrasive media–workpiece wall interaction interface. (**a**) Macroscale representation of the interface between the abrasive media and the workpiece wall. Microscopic details of the SAM and BAM interaction interfaces are shown in (**b**,**c**), respectively.

To understand the underlying cause of this observation, we turned our attention to the slip layer model. Figure 10 offers an intricate visualization of the slip mechanism occurring at the interface between the abrasive media and the workpiece wall. As illustrated in Figure 10a, from a macro perspective, the slip layer model theorizes that at the liquid-solid juncture, shear forces, and the relatively diminished viscosity of the lubricant (compared to the polymer matrix) push the lubricant to migrate to the wall, forming a svelte, which is a continuous slip layer [28,40,41,42,43]. It is noteworthy that the abrasive media combine components such as polymer matrixes, plasticizers, abrasive particles, and lubricants. Moreover, plasticizers gel harmoniously with the polymer matrix, and lubricants stand in contrast, evidencing a lack of adhesion between them. This unique characteristic of lubricants, combined with their inherent slipperiness, results in the lubricant sliding over the solid interface, which minimizes friction [44]. This situation clearly shows the feasibility of the slip layer mechanism, especially when the abrasive medium is infused with a lubricant.

Diving deeper into the amount of additives in SAM and BAM, a complex interaction behavior with the interfacing surface emerges. During the preparation of the abrasive media, we incorporated a higher proportion of the lubricant in BAM (as detailed in Table 1). As visualized in Figure 10b, such a formulation might generate a more pronounced migration of the lubricant towards the wall when subjected to shearing, resulting in a thicker slip layer. This enlarged layer then reduces the contact surface and the adhesive forces between the wall and the abrasive media, enhancing the wall slip intensity. The origins of our hypothesis trace back to, and find corroboration in, the insights shared by Scarratt et al. [45]. It is worth noting that even though the magnitude of the slip layer’s thickness (which typically lies within the micrometer range [14]) is considerably smaller than the channel dimensions and the composition ratio inside the abrasive media remains fairly consistent, we can still decompose the overall flow into distinct segments comprising wall slip flow and internal shear flow, as illustrated in Figure 2b.

This discovery holds significant implications for our understanding of how the wall slip behavior of abrasive media affects the AFM polishing outcome. For instance, exploring and optimizing slip characteristics might pave the way for enhanced polishing results, especially at the micro- and nanoscales.

### 4.2. Rheological Properties and Characterization

This section delves into the rheological attributes of the two abrasive media, SAM and BAM. Our investigation begins with an exploration of their dynamic mechanical properties, specifically under varying frequencies. Subsequently, we explore the intricacies of wall slip under CSRT, dissect its broader implications, and evaluate the validity of the Cox–Merz rule in this context. Following this, we will derive the relationship between wall shear rate and shear viscosity based on the findings from CFT, applying a corrective formula. Finally, we will employ various rheological models to describe the shear viscosity–shear rate relationship, aiming to offer a more comprehensive characterization of the flow properties inherent to these abrasive media.

#### 4.2.1. Linear Viscoelastic Properties

**Figure 11:**
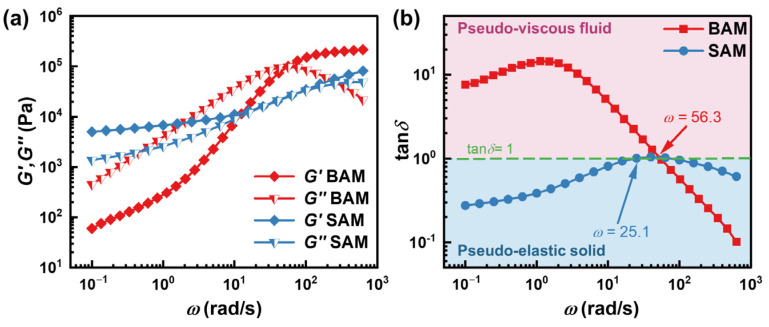
Logarithmic representations of (**a**) storage and loss modulus and (**b**) loss factor, plotted against angular frequency from SAOS measurements for SAM at 1% strain amplitude and 20 °C, and for BAM at 0.1% strain amplitude and 15 °C.

Figure 11 showcases the dynamic mechanical behavior of SAM and BAM. For SAM, both the loss modulus and storage modulus increase with the rise in frequency. The loss factor exceeds 1 only in the frequency range of 23–75 rad/s, suggesting that SAM behaves like a viscoelastic solid. This phenomenon can potentially be attributed to SAM’s foundational composition, derived from hydrocarbon oil-infused SBR.

In contrast, BAM’s behavior demonstrates a different pattern. The loss factor in BAM exceeds 1 for frequencies below 56.3 rad/s, implying the liquid-like tendencies of this abrasive media. Subsequent to this frequency threshold, the loss modulus undergoes a steep decline, while the storage modulus observes a slight increase before plateauing. In the high-frequency segment, the loss factor consistently remains below 1, signifying a pronounced solid-like characteristic inherent to the abrasive media. Moreover, in this high-frequency range, BAM exhibits a higher storage modulus than SAM, suggesting a stronger elasticity in BAM, which might be related to its composition and structural characteristics. This elasticity could potentially elucidate the observations in Section 4.1.1, where BAM exhibited higher entrance pressure losses. This is conceivable, as the abrasive media navigates intense shear and elongation during its trajectory through the capillary inlet, culminating in pronounced energy losses attributable to both elastic storage and frictional losses.

#### 4.2.2. Examination of the Applicability of the Cox–Merz Rule

**Figure 12:**
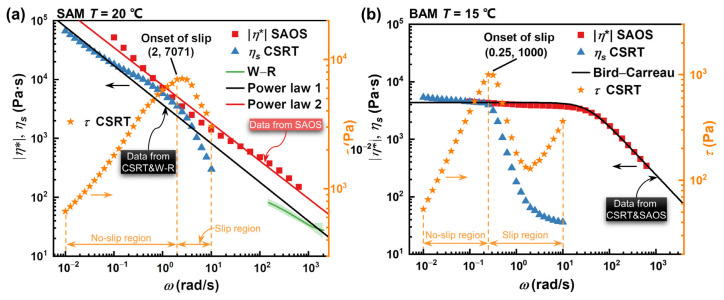
Log–log plots of rheological properties, Cox–Merz rule applicability, and constitutive model fitting for (**a**) SAM and (**b**) BAM. The plots exhibit the correlations of complex viscosity with angular frequency derived from SAOS tests, shear viscosity with shear rate from CSRT, and the association of shear viscosity for SAM, corrected by W−R, with shear rate from capillary flow tests (depicted as a green line plot with error bands). Power-law and cross-law model fittings are applied to SAM and BAM, respectively. The right y-axis shows the relationship between shear stress and shear rate obtained from CSRT.

Utilizing the wall slip velocity data (as illustrated in Figure 9) and wall shear stress data (from Figure 7a) from CFT, we corrected the true wall shear rate-shear viscosity relationship using Equation (7). This approach offers a method to gauge the shear viscosity in regions with high shear rates, specifically for the abrasive media that deviate from the Cox–Merz rule. Figure 12 aggregates the rheological evaluations of both abrasive media—SAM and BAM—sourced from various measurement methodologies. This includes the relationship dynamics between shear stress vs. shear rate from CSRT, the complex viscosity vs. frequency derived from SAOS, and the shear viscosity vs. shear rate extracted from CFT.

The results reveal that both SAM and BAM exhibit intricate flow behaviors at different shear rates and frequencies. Primarily, it is evident that both abrasive media display shear-thinning tendencies during shearing, characterized by a decline in shear viscosity with increasing shear rates—a phenomenon that aligns with conclusions drawn in previous studies [30,37,38,46]. Furthermore, our observations under CSRT indicate that, upon reaching or surpassing a specific shear rate threshold (2.0 s^−1^ for SAM and 0.25 s^−1^ for BAM), there is a sharp decline in both shear stress and shear viscosity. This is consistent with earlier studies [20,29,30]. Such behavior can be traced back to the wall slip phenomenon, where, under high shear stresses, if the sample cannot sustain the applied shear stress, it succumbs to slippage on the fixture interface. This results in an actual shear stress inferior to the anticipated values, leading to a sudden drop in measured viscosity.

We now focus on evaluating the applicability of the Cox–Merz rule. This empirical rule offers a bridge between a fluid’s shear viscosity and its complex viscosity. For SAM, it was observed that its complex viscosity exceeded its shear viscosity before slip onset, suggesting that the Cox–Merz rule might not be applicable. On the other hand, BAM displayed a nearly identical complex and shear viscosity prior to the onset of slip, coupled with closely matched shear-thinning slopes. This provides evidence supporting the validity of the Cox–Merz relationship for BAM.

#### 4.2.3. Rheological Characterization

Upon closer examination of SAM’s CFT data, it became evident that the shear viscosity, after W−R correction, was consistent with the non-slip region shear viscosity trend from CSRT. This further underlines the shear-thinning nature. Thus, by integrating the W−R corrected viscosity with CSRT-derived viscosity, we can aptly capture the shear viscosity properties of the abrasive media such as SAM, which deviate from the Cox–Merz rule. The observed continuous shear-thinning behavior without any discernible Newtonian plateau drove us to adopt the power-law model (refer to Equation (A2) in Appendix A) for a fitting description. The curve fitting of the shear viscosity–shear rate relationship, shown as the black line in Figure 12a, yielded the constitutive parameters listed in Table 3. With an impressive R^2^ value of 0.989, the Power model appears to be a fitting choice. To provide comparative validation against the findings from Section 5, the SAOS viscosity results were also fitted using the power-law model, which is depicted by the red line in Figure 12a. This allowed for a comparison between different rheological characterization techniques and their ability to predict experimental results.

BAM, however, posed certain challenges. Notably, its wall slip velocity closely mirrored the average fluid velocity, leading to significant discrepancies in the W−R corrected viscosity across varying diameters. This also resulted in physically implausible negative wall shear rates, suggesting that the W−R correction is less effective with extremely high wall slip velocities. These observations strongly indicate that the W−R correction might struggle in scenarios with exceptionally high wall slip velocities. Rather than a fundamental flaw of the method, this issue seems to arise from its sensitivity to experimental errors and data processing under such conditions. As a result, we chose not to display these outcomes in Figure 12b.

Nevertheless, despite the complications surrounding the W−R viscosity correction for BAM, the adherence of BAM to the Cox–Merz rule allowed us to determine its shear viscosity over a shear rate spectrum of 0.01–618 s^−1^. This was accomplished using both CSRT and SAOS data. Interestingly, BAM exhibited a minimal decline in viscosity at lower shear rates, suggesting that it behaves like a near-Newtonian fluid. This observation suggests that BAM’s viscosity remains relatively consistent at low shear stresses. Given this behavior, the Bird–Carreau model (refer to Equation (A4) in Appendix A) was selected to depict its flow dynamics. By fitting the shear viscosity–shear rate relationship, we derived the constitutive parameters, which are detailed in Table 3. The fit curve in Figure 12b aligns well with the experimental data, with an R^2^ value of 0.925, confirming the efficacy of the Bird–Carreau model.

## 5. Validation of the Rheological and Wall Slip Models

This section focuses on validating the rheological and wall slip models. Our aim is to understand the impact of various rheological characterization methodologies and wall slip conditions (both in the presence and absence of slip) on the accuracy of entrance pressure predictions.

For validation purposes, we utilized a tube with specifications of 12 mm in diameter and 336 mm in length. Every result was derived from the mean of three repeated measurements, which covered the entrance pressure and the corresponding apparent shear rate.

In our prediction framework, we divided the projected entrance pressure into two facets: the pressure drop in the fully developed zone and the entrance pressure loss. As Figure 7b highlights, at a consistent apparent shear rate, the entrance pressure loss remains relatively independent of diameter variations. This observation aligns with the findings from a study by Karapetsas and Mitsoulis [47], which indicated that, in systems with a contraction ratio (i.e., barrel diameter to tube diameter) between 7.5 and 18.75, the entrance pressure loss is barely influenced by the contraction ratio. Leveraging this insight, we used cubic B-spline interpolation to calculate the mean entrance pressure loss. This interpolated value was subsequently used to estimate entrance pressure loss during validation experiments.

To predict the pressure drop in the fully developed region, an analytical connection between wall shear stress and volumetric flow rate was established for various constitutive models in the capillary flow’s fully developed zone. These models and their associated numerical solutions are elaborated upon in Appendix A. Following this, and using Equation (1), we calculated the pressure drop in this region. Combining this with the entrance pressure loss for the same apparent shear rate provided the full model predictions.

**Figure 13:**
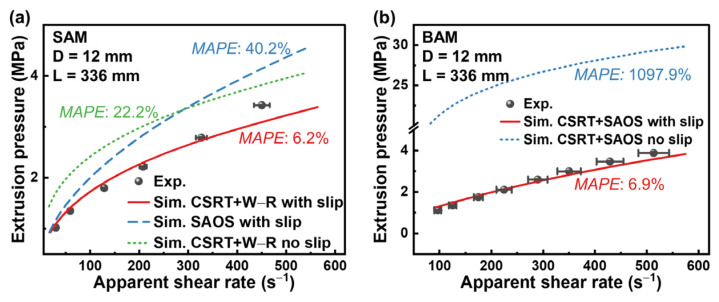
Comparative analysis of the predicted and experimental entrance pressure for (**a**) SAM and (**b**) BAM using diverse rheological characterizations and wall slip boundary conditions. The data points marked by black spheres, accompanied by error bars, reflect experimental results, while the continuous lines represent the predictions. Additionally, the *MAPE* between the experimental and predicted values is presented.

To quantify prediction discrepancies, we adopted the mean absolute percentage error (MAPE) formula, expressed as

(12) $$MAPE=\frac{100\%}{N}\sum_{i=1}^{N}\left|\frac{y_{\mathrm{true},i}-y_{\mathrm{pred},i}}{y_{\mathrm{true},i}}\right|$$

where N denotes the total data points, $y_{\mathrm{true},i}$ the observed values, and $y_{\mathrm{pred},i}$ the model’s predictions. Figure 13 contrasts the experimental extrusion pressures with the model predictions for both SAM and BAM, incorporating various rheological characterization techniques and wall slip conditions.

For SAM, with W−R viscosity adjustments and wall slip considered, the model’s predictions are in close harmony with the experimental results, manifesting a *MAPE* of a mere 6.2%. Conversely, directly applying the SAOS results for constitutive equation characterization results in a higher error of 22.2%. Ignoring wall slip further exacerbates this error, pushing it to 40.2%. In BAM’s case, considering wall slip achieves a *MAPE* of just 6.9%. However, sidestepping wall slip boosts this error dramatically to 1097.9%. This sharp rise makes sense, considering BAM’s stronger tendency to slip. By neglecting wall slip, the pressure drop prediction in the fully developed region deviates significantly from the observed values.

To summarize, these findings emphasize the necessity of validating the Cox–Merz rule’s suitability when examining the rheological properties of the abrasive media. They also highlight the key role of wall slip conditions in making accurate flow predictions for AFM. It is pivotal to acknowledge that, at times, there exists a divergence between model predictions and actual experimental outcomes. This discrepancy can be rooted in the intricate nature of experimental setups or certain oversimplifications inherent in the model’s assumptions. Nevertheless, the overall model validation outcomes confirm the efficacy of the rheological and wall slip characterization methods suggested in this study. This offers a powerful tool to delve deeper into the behavior and predictability of the abrasive media in AFM operations.

## 6. Conclusions

This ground-breaking study aims to elucidate the subtleties and complexities within the AFM field, with a focus on developing a comprehensive rheological and wall slip measurement framework for the abrasive media at elevated shear rates, beyond the critical shear rate at which wall slip onset occurs. The development of the Bagley-corrected Mooney method based on capillary flow and its measurement tools exemplifies the innovative approach taken to bridge the gap between theoretical understanding and experimental observations.

The key findings of this research are as follows:

(1) The abrasive media exhibit Navier nonlinear wall slip, as successfully revealed with the Mooney method. Drawing from the literature, the formation of a lubricant layer, influenced by internal shear forces, might contribute to this slip. The lubricant concentration could be a pivotal factor in this behavior.
(2) CFT analysis uncovers significant wall slip phenomena and entrance pressure loss effects. The findings underscore the importance of our proposed compensation correction strategy for accurate evaluations. Through meticulous analysis of rheological data, the enhanced CFT method proved effective, especially for determining the shear viscosity of the abrasive media not aligned with the Cox–Merz rule. This is especially important at shear rates beyond the critical wall slip inception point, which are typically within 10 s^−1^.
(3) Two fundamental models, the tailored constitutive model and the slip model, are effectively developed through the comprehensive framework. The first captures the inherent rheological attributes of the abrasive media, while the second details the interaction dynamics between the abrasive media and the solid boundary. This duality of insights culminates in the formulation of analytical prediction models tailored for the fully developed capillary flow of the abrasive media. The MAPE between the experimental data and predicted outcomes, which does not exceed 6.9%, demonstrates the models’ remarkable fidelity under careful scrutiny. Such close congruence demonstrates the comprehensive framework’s robustness, accuracy, and practicality.

The results of this study provide substantial support for authentic modeling and material removal predictions. However, a limitation of this study is that it did not consider the impact of wall roughness on wall slip behavior. Future research should delve deeper into the components of the abrasive media, such as abrasive particle concentration and size, and their effects on wall slip, as well as how wall slip behavior influences the polishing outcomes in AFM.

## Figures and Tables

**Table 1:** Detailed parameters of abrasive media.

Parameters	SAM	BAM
Matrix	SBR	BSR
Abrasive particle	SiC 80#	SiC 80#
Volume fraction of abrasive particles	18.95 vol%	18.95 vol%
Mass fraction of abrasive particles	45.00 wt%	43.95 wt%
Mass fraction of lubricant	2.93 wt%	9.19 wt%
Density of abrasive media	1.35 g/cm^3^	1.38 g/cm^3^

**Table 2:** Comparative analysis and fitting results of Mooney method, based on different wall slip models.

Parameters	SAM		BAM	
	Model 1	Model 2	Model 1	Model 2
$k_{s}\;(\mathrm{mm}/s\cdot {\mathrm{Pa}}^{-m_{s}})$	1.84 × 10^−2^	4.565 × 10^−6^	4.60 × 10^−2^	2.953 × 10^−4^
$m_{s}$	1	1.796	1	1.493
R^2^	0.881	0.997	0.924	0.991
AIC	401.27	168.91	231.10	117.84
BIC	405.42	175.04	234.88	123.39
Preferred model	Model 2		Model 2	

**Table 3:** Compilation of the constitutive parameters derived from least-squares fitting of the shear viscosity versus shear rate curves for the abrasive media under consideration.

Abrasive Media	Data Source	Power-Law Model		Bird–Carreau Model		
		K ($\mathrm{Pa}\cdot s^{n}$)	n	$\eta_{0}$ (Pa·s)	λ (s)	n
SAM	CSRT & W−R	3522.739	0.333	-	-	-
	SAOS	7948.124	0.359	-	-	-
BAM	CSRT & SAOS	-	-	4330.660	0.029	0.144

-: None.

## Data Availability

Not applicable.

## Supplementary Materials

The following supporting information can be downloaded at: https://www.mdpi.com/article/10.3390/ma16206803/s1, Table S1: Extrusion pressure and apparent shear rate data for SAM with a capillary diameter of 4 mm. Table S2: Extrusion pressure and apparent shear rate data for SAM with a capillary diameter of 6 mm. Table S3: Extrusion pressure and apparent shear rate data for SAM with a capillary diameter of 8 mm. Table S4: Extrusion pressure and apparent shear rate data for BAM with a capillary diameter of 4 mm. Table S5: Extrusion pressure and apparent shear rate data for BAM with a capillary diameter of 6 mm. Table S6: Extrusion pressure and apparent shear rate data for BAM with a capillary diameter of 8 mm. Figure S1: Bagley correction plots comparing the flow behavior of SAM and BAM in a capillary with a diameter of 4 mm.

## Nomenclature and Abbreviations

**Nomenclature**		$y_{\mathrm{true},i}$ [−]	Actual observed values from experiments
D [m]	Diameter of circular tube	$y_{\mathrm{pred},i}$ [−]	Predicted values
$D_{b}$ [m]	Diameter of abrasive cylinder	γ [−]	Shear strain
$G^{\prime }$ [Pa]	Storage modulus	$\gamma_{0}$ [−]	Maximum value of oscillating shear strain
$G^{\prime \prime }$ [Pa]	Loss modulus	$\dot{\gamma }$ [s^−1^]	Shear rate
$k_{s}$ [$\mathrm{mm}/s\cdot {\mathrm{Pa}}^{-m_{s}}$]	Slip coefficient	${\dot{\gamma }}_{a}$ [s^−1^]	Apparent shear rate
K [Pa·s^n^]	Consistency index	${\dot{\gamma }}_{0}$ [s^−1^]	Maximum value of oscillating shear rate
L [m]	Length of circular tube	${\dot{\gamma }}_{w}$ [s^−1^]	The shear rate at the wall
$m_{s}$ [−]	Slip index	$\eta_{s}$ [Pa·s]	Shear viscosity
MAPE [−]	Mean absolute percentage error	$\eta_{0}$ [Pa·s]	Viscosity at zero shear rate
n [−]	Power-law index	$\eta_{\infty }$ [Pa·s]	Viscosity at infinite shear rate
N[−]	Total number of observations	$\left|\eta^{*}\right|$ [Pa·s]	Complex viscosity
p [Pa]	Pressure	λ [s]	Time constant
$p_{b}$ [Pa]	Extrusion pressure	$\tau_{rz}$ [Pa]	Shear stress acting in the z-direction on a plane normal to the r-direction
$\Delta p_{en}$ [Pa]	Entrance pressure drop	$\tau_{w}$ [Pa]	Shear stress on the wall
$\Delta p_{fu}$ [Pa]	Pressure drop in fully developed region	ω [rad/s]	Frequency of oscillation
$\Delta p_{ex}$ [Pa]	Exit pressure drop	**Abbreviations**	
Q [m^3^/s]	Volume flow rate	AIC	Akaike information criterion
$Q_{shear}$, Γ(r) [m^3^/s]	Shear flow contribution to volume flow rate	AFM	Abrasive flow machining
$Q_{slip}$ [m^3^/s]	Wall slip contribution to volume flow rate	BAM	BSR-based abrasive media
r, θ, z [m, rad, m]	Coordinates of a cylindrical coordinate system	BIC	Bayesian information criterion
R [m]	Radius of circular tube	BSR	Boron-modified silicone rubber
s [m]	Displacement of piston	CFT	Capillary flow test
t [s]	Time	CSRT	Controlled shear rate test
tanδ [−]	Loss factor	SAM	SBR-based abrasive media
T [°C]	Temperature	SAOS	Small amplitude oscillatory shear
$v_{z}$ [m/s]	Velocity component in the z-direction	SBR	Styrene-butadiene rubber
$v_{w}$ [m/s]	Velocity at wall	W−R	Weissenberg–Rabinowitsch

## Appendix A. Analytical Models for Power-Law and Bird–Carreau Capillary Flows

To elucidate the relationship between $\tau_{w}$ and Q(R) as expressed in Equation (3), it is essential to detail the correlation between $\tau_{rz}$ and $\dot{\gamma }$, known as the constitutive model. This correlation is fundamental for computing the integral function $\Gamma (\tau_{w})$. For the rheological properties of the abrasive media addressed in this study, two specific viscous models were employed: the power-law model and the Bird–Carreau model. The power-law model offers a simplified yet insightful perspective, capturing many non-Newtonian fluid characteristics. In contrast, the Bird–Carreau model provides a more comprehensive representation, accounting for both Newtonian and non-Newtonian behaviors under different shear rate conditions.

Within the confines of pure viscous models, the shear stress’s relationship with shear rate can be expressed as shown in Equation (A1) as follows:

(A1) $$\tau_{rz}=\eta_{s}\dot{\gamma }$$

For the power-law model applied to SAM, viscosity $\eta_{s}$ and shear rate $\dot{\gamma }$ follow the relationship of

(A2) $$\eta_{s}=K{\dot{\gamma }}^{n-1}$$

where K refers to the consistency coefficient, and n represents the power-law index. The integral function can be derived by substituting Equations (A2) and (A1) into the second term on the right side of Equation (3), resulting in a simplified form as follows:

(A3) $$\Gamma (\dot{\gamma }(r))=\frac{\pi R^{3}K^{3}}{\tau_{w}{}^{3}(3+1/n)}\dot{\gamma }{\left(r\right)}^{3n+1}$$

Considering the Bird–Carreau model applied to BAM, if the infinite shear rate viscosity $\eta_{\infty }$ equals 0, the constitutive equation is obtained with

(A4) $$\eta_{s}=\eta_{0}{\left(1+\lambda^{2}{\dot{\gamma }}^{2}\right)}^{(n-1)/2}$$

In this equation, λ denotes the characteristic time constant (the reciprocal of the shear rate at the transition from Newtonian to power-law behavior), n signifies the power-law index, and $\eta_{0}$ corresponds to the zero-shear viscosity. The integral function is calculated by combining Equations (A1) and (A4) and making a substitution into the second term on the right side of Equation (3), yielding a simplified result, as follows:

(A5) $$\Gamma (\dot{\gamma }(r))=\frac{\pi R^{3}\eta_{0}{}^{3}}{\tau_{w}^{3}\lambda^{4}}\frac{{\left({\dot{\gamma }}^{2}\lambda^{2}+1\right)}^{3(n-1)/2}[(9n^{2}-3n){\dot{\gamma }}^{4}\lambda^{4}-3n{\dot{\gamma }}^{2}\lambda^{2}+3{\dot{\gamma }}^{2}\lambda^{2}+2]-2}{\left(3n-1)(9n+3\right)}$$

To calculate the wall shear stress–volume flow rate relationship, we employed Matlab (version 2018b) commercial software to obtain the numerical solutions. First, the methodology involved defining the wall shear stress in the fully developed region; then, it involved combining Equation (A1) with the abrasive media’s constitutive equation to resolve the wall shear rate. Subsequently, we input this wall shear rate into the corresponding integral function’s analytical expression (either Equation (A3) or (A5)) to determine the shear flow-contributed volume flow rate. Lastly, we computed the wall slip model-derived plunger flow volume rate (as per Equation (11)) and added it to the shear flow-contributed rate to achieve the total volume flow rate.
