# Supplementary material for: On Characterization of Shear Viscosity and Wall Slip for Concentrated Suspension Flows in Abrasive Flow Machining
Source: Materials (Basel). 2023 Oct 22;16(20):6803. doi: 10.3390/ma16206803 (PMC10608499; doi:10.3390/ma16206803)
Supplement: Supplementary file 1 [file materials-16-06803-s001.zip › materials-2653261-supplementary.pdf]

# Supplementary

Table S1. Extrusion pressure and apparent shear rate data for SAM with a capillary diameter of 4 mm.

| $L/D=20$ | | $L/D=30$ | | $L/D=40$ | |
|---|---|---|---|---|---|
| $\dot{\gamma}_a$ (s$^{-1}$) | $p_b$ (MPa) | $\dot{\gamma}_a$ (s$^{-1}$) | $p_b$ (MPa) | $\dot{\gamma}_a$ (s$^{-1}$) | $p_b$ (MPa) |
| 149.73±8.52 | 1.01±0.00 | 64.99±9.20 | 0.99±0.00 | 20.00±5.20 | 1.01±0.00 |
| 272.62±12.73 | 1.34±0.00 | 140.46±9.50 | 1.35±0.00 | 49.52±6.80 | 1.34±0.01 |
| 465.51±22.97 | 1.74±0.00 | 269.71±13.42 | 1.79±0.01 | 177.31±10.61 | 1.82±0.01 |
| 794.24±37.04 | 2.34±0.01 | 446.00±14.90 | 2.37±0.01 | 258.00±13.80 | 2.34±0.01 |
| 1275.66±58.57 | 2.77±0.01 | 611.15±25.61 | 2.75±0.01 | 345.31±19.69 | 2.74±0.01 |
| 1861.87±79.72 | 3.44±0.01 | 945.97±33.18 | 3.52±0.01 | 649.38±28.98 | 3.53±0.01 |
| 2918.68±126.36 | 4.32±0.01 | 1623.32±66.20 | 4.42±0.02 | 860.64±41.27 | 4.39±0.02 |
| 3798.90±156.03 | 4.83±0.02 | 1890.02±89.86 | 4.87±0.01 | 1072.34±51.30 | 4.90±0.02 |
| - | - | 2314.81±129.39 | 5.36±0.01 | 1400.20±45.86 | 5.37±0.01 |
| - | - | 2751.11±143.99 | 5.95±0.10 | 1681.20±78.64 | 6.00±0.02 |
| - | - | 3589.52±159.51 | 6.78±0.01 | 2263.21±79.06 | 6.86±0.01 |
| - | - | - | - | 2690.69±98.04 | 7.78±0.01 |
| - | - | - | - | 3529.87±129.11 | 8.87±0.01 |

Table S2. Extrusion pressure and apparent shear rate data for SAM with a capillary diameter of 6 mm.

| $L/D=20$ | | $L/D=30$ | | $L/D=40$ | |
|---|---|---|---|---|---|
| $\dot{\gamma}_a$ (s$^{-1}$) | $p_b$ (MPa) | $\dot{\gamma}_a$ (s$^{-1}$) | $p_b$ (MPa) | $\dot{\gamma}_a$ (s$^{-1}$) | $p_b$ (MPa) |
| 133.82±5.41 | 1.12±0.00 | 48.25±8.85 | 1.08±0.00 | 40.12±8.21 | 1.11±0.00 |
| 196.25±15.89 | 1.32±0.00 | 80.11±11.25 | 1.34±0.00 | 59.13±8.52 | 1.38±0.00 |
| 332.24±18.13 | 1.77±0.00 | 189.89±17.03 | 1.81±0.00 | 84.90±9.13 | 1.80±0.00 |
| 631.38±28.10 | 2.20±0.00 | 282.54±16.46 | 2.28±0.01 | 179.83±9.64 | 2.19±0.00 |
| 965.11±43.11 | 2.77±0.01 | 493.71±25.24 | 2.77±0.01 | 278.79±14.44 | 2.81±0.00 |
| 1496.69±77.81 | 3.38±0.02 | 711.91±32.65 | 3.42±0.01 | 430.30±22.14 | 3.41±0.01 |
| 2358.39±122.42 | 4.16±0.01 | 1121.85±48.50 | 4.28±0.00 | 687.64±30.82 | 4.26±0.01 |
| 2883.84±139.16 | 4.71±0.01 | 1400.37±64.09 | 4.74±0.01 | 887.09±30.62 | 4.82±0.01 |
| - | - | 1891.11±80.21 | 5.49±0.01 | 1099.31±43.96 | 5.45±0.01 |
| - | - | 2272.96±110.05 | 5.99±0.01 | 1328.64±60.40 | 5.89±0.01 |
| - | - | 2807.78±138.05 | 6.79±0.01 | 1856.18±96.97 | 6.88±0.01 |
| - | - | - | - | 2196.75±100.15 | 7.79±0.01 |
| - | - | - | - | 2821.60±136.76 | 8.61±0.02 |

Table S3. Extrusion pressure and apparent shear rate data for SAM with a capillary diameter of 8 mm.

| $L/D=20$ | | $L/D=30$ | | $L/D=40$ | |
|---|---|---|---|---|---|
| $\dot{\gamma}_a$ ($s^{-1}$) | $p_b$ (MPa) | $\dot{\gamma}_a$ ($s^{-1}$) | $p_b$ (MPa) | $\dot{\gamma}_a$ ($s^{-1}$) | $p_b$ (MPa) |
| 79.84±5.58 | 1.05±0.01 | 38.55±3.50 | 1.01±0.01 | 22.13±3.10 | 0.99±0.00 |
| 169.38±6.27 | 1.35±0.01 | 70.13±4.20 | 1.32±0.01 | 38.98±4.50 | 1.35±0.01 |
| 289.56±16.26 | 1.74±0.01 | 156.76±5.26 | 1.80±0.02 | 75.25±6.20 | 1.80±0.01 |
| 486.04±21.52 | 2.25±0.02 | 245.37±11.71 | 2.21±0.00 | 156.21±7.92 | 2.25±0.01 |
| 788.14±36.36 | 2.78±0.01 | 416.30±11.89 | 2.77±0.01 | 237.94±12.59 | 2.79±0.01 |
| 1153.58±46.22 | 3.41±0.01 | 580.27±15.17 | 3.46±0.01 | 357.53±17.78 | 3.40±0.01 |
| 1858.22±58.49 | 4.29±0.01 | 939.78±25.60 | 4.29±0.01 | 550.58±22.80 | 4.31±0.01 |
| - | - | 1181.33±61.42 | 4.79±0.02 | 683.68±30.39 | 4.80±0.02 |
| - | - | 1495.80±64.71 | 5.38±0.03 | 888.12±46.80 | 5.44±0.02 |
| - | - | 1759.40±80.75 | 5.92±0.01 | 1073.06±45.44 | 6.07±0.04 |
| - | - | - | - | 1434.69±68.44 | 6.80±0.02 |
| - | - | - | - | 1731.19±84.95 | 7.82±0.04 |

Table S4. Extrusion pressure and apparent shear rate data for BAM with a capillary diameter of 4 mm.

| $L/D=20$ | | $L/D=30$ | | $L/D=40$ | |
|---|---|---|---|---|---|
| $\dot{\gamma}_a$ ($s^{-1}$) | $p_b$ (MPa) | $\dot{\gamma}_a$ ($s^{-1}$) | $p_b$ (MPa) | $\dot{\gamma}_a$ ($s^{-1}$) | $p_b$ (MPa) |
| 202.50±11.54 | 1.09±0.01 | 170.85±9.37 | 1.10±0.01 | 145.53±7.08 | 1.10±0.01 |
| 274.47±14.33 | 1.30±0.01 | 220.62±9.43 | 1.34±0.01 | 187.07±10.36 | 1.34±0.01 |
| 419.34±24.19 | 1.73±0.01 | 373.87±15.64 | 1.77±0.01 | 304.68±16.76 | 1.78±0.01 |
| 616.98±31.70 | 2.13±0.02 | 503.07±29.27 | 2.14±0.01 | 406.52±20.01 | 2.20±0.01 |
| 983.80±40.20 | 2.75±0.04 | 699.65±35.05 | 2.71±0.01 | 545.62±27.18 | 2.77±0.01 |
| 1273.66±66.45 | 3.47±0.02 | 984.90±43.99 | 3.41±0.02 | 766.07±41.24 | 3.44±0.02 |
| 1681.70±88.21 | 4.03±0.03 | 1335.54±67.38 | 4.15±0.02 | 1116.10±60.26 | 4.23±0.02 |
| 2162.80±100.48 | 4.48±0.05 | 1555.69±88.06 | 4.65±0.03 | 1247.51±64.22 | 4.76±0.02 |
| 2679.78±130.28 | 5.21±0.02 | 1887.13±107.38 | 5.18±0.02 | 1432.45±71.99 | 5.29±0.02 |
| 3357.72±160.60 | 5.70±0.03 | 2075.92±128.15 | 5.70±0.02 | 1807.86±70.35 | 5.86±0.02 |
| - | - | 2447.66±120.85 | 6.30±0.00 | 2059.98±121.18 | 6.35±0.03 |
| - | - | 3209.23±148.66 | 7.10±0.03 | 2335.99±118.92 | 7.17±0.03 |
| - | - | 3863.63±147.44 | 7.95±0.06 | 3016.87±101.78 | 8.01±0.05 |
| - | - | - | - | 3556.55±159.32 | 9.12±0.06 |

Table S5. Extrusion pressure and apparent shear rate data for BAM with a capillary diameter of 6 mm.

| $L/D=20$ | | $L/D=30$ | | $L/D=40$ | |
|---|---|---|---|---|---|

| $\dot{\gamma}_a$ (s$^{-1}$) | $p_b$ (MPa) | $\dot{\gamma}_a$ (s$^{-1}$) | $p_b$ (MPa) | $\dot{\gamma}_a$ (s$^{-1}$) | $p_b$ (MPa) |
|---|---|---|---|---|---|
| 155.34±7.00 | 1.01±0.01 | 125.64±6.02 | 1.05±0.01 | 111.54±6.20 | 1.08±0.00 |
| 203.31±13.28 | 1.35±0.01 | 176.39±6.00 | 1.35±0.01 | 142.50±6.56 | 1.36±0.04 |
| 357.56±16.98 | 1.76±0.01 | 235.13±14.13 | 1.71±0.01 | 200.30±7.58 | 1.68±0.01 |
| 462.24±26.89 | 2.16±0.01 | 341.48±19.37 | 2.15±0.01 | 303.45±13.00 | 2.21±0.04 |
| 691.57±37.28 | 2.71±0.01 | 513.24±24.71 | 2.71±0.01 | 377.65±20.12 | 2.74±0.03 |
| 959.48±66.85 | 3.36±0.04 | 731.67±25.50 | 3.35±0.02 | 576.07±26.16 | 3.42±0.03 |
| 1372.07±66.51 | 4.01±0.02 | 967.05±39.52 | 4.07±0.03 | 810.34±31.37 | 4.21±0.03 |
| 1508.88±95.74 | 4.54±0.05 | 1209.63±63.40 | 4.78±0.04 | 1039.09±51.00 | 5.02±0.05 |
| 2004.49±109.94 | 5.13±0.07 | 1555.19±54.95 | 5.48±0.12 | 1199.82±59.46 | 5.67±0.03 |
| 2319.97±119.34 | 5.71±0.11 | 1966.65±120.12 | 6.28±0.13 | 1457.50±71.98 | 6.34±0.06 |
| 2745.20±135.28 | 6.32±0.15 | 2301.64±139.12 | 7.25±0.12 | 1808.44±109.10 | 7.36±0.03 |
| - | - | 2919.99±146.70 | 8.17±0.15 | 2112.23±101.44 | 8.33±0.12 |
| - | - | - | - | 2614.28±139.01 | 9.51±0.25 |

Table S6. Extrusion pressure and apparent shear rate data for BAM with a capillary diameter of 8 mm.

| $L/D=20$ | | $L/D=30$ | | $L/D=40$ | |
|---|---|---|---|---|---|
| $\dot{\gamma}_a$ (s$^{-1}$) | $p_b$ (MPa) | $\dot{\gamma}_a$ (s$^{-1}$) | $p_b$ (MPa) | $\dot{\gamma}_a$ (s$^{-1}$) | $p_b$ (MPa) |
| 154.37±8.10 | 1.11±0.01 | 137.81±6.90 | 1.12±0.01 | 105.14±7.98 | 1.13±0.01 |
| 221.25±9.77 | 1.35±0.01 | 172.82±8.50 | 1.37±0.01 | 126.99±8.24 | 1.35±0.01 |
| 273.53±13.16 | 1.72±0.01 | 218.80±12.71 | 1.75±0.01 | 179.90±9.75 | 1.75±0.01 |
| 354.63±23.21 | 2.07±0.02 | 260.05±17.36 | 2.10±0.01 | 203.03±22.84 | 2.11±0.02 |
| 497.44±31.31 | 2.60±0.02 | 393.31±25.10 | 2.60±0.02 | 298.36±26.88 | 2.66±0.02 |
| 617.69±31.72 | 2.98±0.02 | 477.26±29.11 | 3.00±0.01 | 416.60±27.64 | 3.01±0.02 |
| 815.50±46.71 | 3.46±0.03 | 608.27±29.36 | 3.50±0.02 | 425.07±32.90 | 3.45±0.03 |
| 973.05±47.82 | 3.88±0.03 | 684.88±40.76 | 3.84±0.05 | 575.20±35.59 | 3.91±0.07 |
| 1212.29±51.04 | 4.18±0.02 | 828.18±41.95 | 4.25±0.02 | 673.27±32.88 | 4.32±0.01 |
| 1354.42±69.40 | 4.75±0.09 | 980.54±42.92 | 4.78±0.01 | 749.58±38.85 | 4.79±0.09 |
| 1583.91±95.91 | 5.11±0.13 | 1098.43±43.86 | 5.09±0.05 | 810.90±30.51 | 5.14±0.05 |
| - | - | 1221.57±65.50 | 5.56±0.13 | 844.27±56.29 | 5.61±0.12 |
| - | - | 1465.15±69.14 | 6.22±0.15 | 1144.89±58.07 | 6.32±0.09 |
| - | - | - | - | 1388.94±68.06 | 7.35±0.12 |
| - | - | - | - | 1680.07±76.01 | 8.43±0.15 |

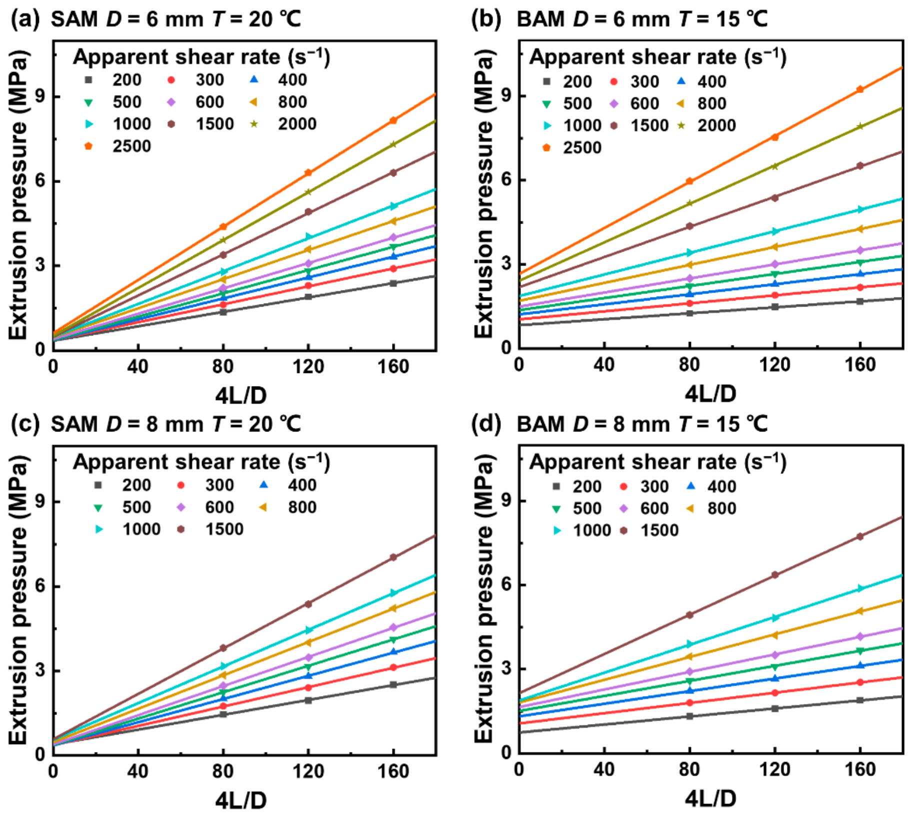


Figure S1. Bagley correction plots elucidating the flow behavior of SAM and BAM in capillaries of varying diameters. Subplots (a) and (c) represent SAM for diameters of 6 mm and 8 mm, respectively. Subplots (b) and (d) illustrate BAM for the same diameters. Data points represent the results obtained from the cubic B-spline interpolation in Figure 6, with differences in color and symbol shape indicating different apparent shear rates.
